# Autophosphorylation and the Dynamics of the Activation of Lck

**DOI:** 10.1007/s11538-021-00900-9

**Published:** 2021-05-01

**Authors:** Lisa Maria Kreusser, Alan D. Rendall

**Affiliations:** 1 Department for Applied Mathematics and Theoretical Physics, University of Cambridge, Wilberforce Road, Cambridge, CB3 0WA UK; 2 Institut für Mathematik, Johannes Gutenberg-Universität, Staudingerweg 9, 55099 Mainz, Germany

**Keywords:** Src kinase, Bifurcation, Phosphorylation, Dynamics, T cells

## Abstract

Lck (lymphocyte-specific protein tyrosine kinase) is an enzyme which plays a number of important roles in the function of immune cells. It belongs to the Src family of kinases which are known to undergo autophosphorylation. It turns out that this leads to a remarkable variety of dynamical behaviour which can occur during their activation. We prove that in the presence of autophosphorylation one phenomenon, bistability, already occurs in a mathematical model for a protein with a single phosphorylation site. We further show that a certain model of Lck exhibits oscillations. Finally, we discuss the relations of these results to models in the literature which involve Lck and describe specific biological processes, such as the early stages of T cell activation and the stimulation of T cell responses resulting from the suppression of PD-1 signalling which is important in immune checkpoint therapy for cancer.

## Introduction

Phosphorylation and dephosphorylation, the processes in which proteins are modified by the addition or removal of phosphate groups, play an important role in biology. The activity of an enzyme is influenced by its phosphorylation state, and these processes provide a way of switching enzymes on and off quickly. The enzymes which catalyse phosphorylation and dephosphorylation are called kinases and phosphatases, respectively. The phosphorylation of a protein X is usually catalysed by another protein Y. It may also be catalysed by X itself, a process called autophosphorylation. This can happen either in trans (one molecule of X catalyses the phosphorylation of a site on another molecule of X) or in cis (a molecule of X catalyses the phosphorylation of a site on that same molecule). Here, we are concerned with the kinase Lck (Bommhardt et al. 2019), which can undergo both autophosphorylation in trans and phosphorylation by another kinase Csk. Lck belongs to the Src family of kinases (Shah et al. 2018) which have many properties in common, in particular those related to their phosphorylation.

In what follows we are interested in understanding the way in which the activity of Lck is controlled, an issue which is important for analysing how the function of immune cells is regulated. More specifically, we want to do so by studying mathematical models for phosphorylation processes. There has been a lot of work on models for cases where there is a clear distinction between substrates and enzymes. A standard example is the multiple futile cycle where bounds for the maximal number of steady states were obtained in Wang and Sontag (2008) and Flockerzi et al. (2014) and for the maximal number of stable steady states in Feliu et al. (2020). Much less is known in the case of autophosphorylation. To our knowledge the earliest papers on mathematical modelling of Src family kinases are by Fuß et al. (2006, 2008). In the first of these papers, the authors consider a system coupling Src (with autophosphorylation included) to Csk and the phosphatase PTP$\alpha$. They then introduce a simplification by assuming the concentration of Csk to be constant, and find twofold bifurcations in simulations. In particular, this system appears to exhibit bistability. In Fuß et al. (2008), sustained oscillations and infinite period bifurcations were observed in a slight extension of the model of Fuß et al. (2006). These dynamical features occurred in a context where the basic system describing phosphorylation and dephosphorylation of Src is embedded in feedback loops. In fact, it was found in Kaimachnikov and Kholodenko (2009) that complicated dynamical behaviour is possible even without the feedback loops. More recently the dynamics of a model for autophosphorylation of a protein with only one phosphorylation site was studied in Doherty et al. (2015). In that case, also twofold bifurcations were observed. The model considered there is one-dimensional and thus relatively easy to analyse. The bistability found in Doherty et al. (2015) contrasts with the situation in the multiple futile cycle where in the case of a single phosphorylation site there is only one steady state.

In Sect. 2, a model for autophosphorylation is introduced which is of central importance in what follows and it is shown that in a certain Michaelis–Menten limit it can be reduced to a one-dimensional model. Section 3 contains an analysis of some properties of solutions of this reduced model. In particular, it is shown that this system can exhibit more than one stable steady state. This section provides a rigorous treatment of some features found in the simulations of Doherty et al. (2015). The property of bistability is lifted to the original model. The main results are Theorems 1–3. The model of Sect. 2 without external kinase only exhibits bistability under the condition that phosphorylation has an activating effect on the enzyme. The corresponding case with inhibition exhibits no multistability. The aim of Sect. 4 is to show that in the case of an inhibitory phosphorylation multistability can be restored by modelling the external kinase explicitly. The main result is Theorem 4. Here, in contrast to the results of Sect. 3, the multistability is not present in the Michaelis–Menten limit.

Section 5 is concerned with a model for Lck which can be reduced by timescale separation to a two-dimensional one. The original model inherits certain patterns of behaviour such as bistability, Hopf bifurcations and homoclinic orbits from the two-dimensional one. It is proved that the two-dimensional model does exhibit these phenomena as a consequence of the occurrence of a Bogdanov–Takens bifurcation. The main result is Theorem 5. In Sect. 6, the models analysed in the present paper are compared with ones which occur as parts of more comprehensive models in the literature describing some concrete biological situations. Section 7 presents some ideas on possible further developments of the results of this paper.

## The Basic Model

Consider a protein with one phosphorylation site. We denote the unphosphorylated form of this protein by X and the phosphorylated form by Y. Suppose X is able to catalyse its own phosphorylation in trans. The simplest model for this reaction is 2X$\rightarrow$ X $+$ Y. If Y is also able to catalyse the phosphorylation of X, then this can be modelled by the reaction X $+$ Y $\rightarrow$ 2Y. The basic model considered in what follows includes these two reactions together with phosphorylation of X catalysed by a kinase E and dephosphorylation of Y catalysed by a phosphatase F. Mass action kinetics is assumed for the autophosphorylation reactions in the form written above. For the other two processes, we use a description consisting of elementary reactions involving a substrate, an enzyme and a complex, which we call an extended Michaelis–Menten description. Mass action kinetics is assumed for the elementary reactions. It would be possible to use an alternative description of the autophosphorylation reactions including homo- and heterodimers. We expect that many of the results we obtain could be extended to that more complicated model, but this will not be pursued further here. A further remark on the more complicated alternative can be found in Sect. 6.

With our choices the reaction network is as follows:

$$\begin{aligned} 2\text {X}&\xrightarrow {k_1} \text {X}+ \text {Y}\\ \text {F} + \text {Y}&\xrightarrow {k_2} \text {YF}\\ \text {YF}&\xrightarrow {k_3} \text {Y}+\text {F}\\ \text {YF}&\xrightarrow {k_4} \text {X}+\text {F}\\ \text {E}+\text {X}&\xrightarrow {k_5} \text {XE}\\ \text {XE}&\xrightarrow {k_6} \text {E}+\text {X} \\ \text {XE}&\xrightarrow {k_7} \text {E}+\text {Y} \\ \text {X}+\text {Y}&\xrightarrow {k_8} 2\text {Y}\\ \end{aligned}$$

The concentrations of X, Y, E, F and the complexes XE and YF are denoted by *x*, *y*, *e*, *f*, *d* and *c*, respectively. The evolution equations are of the form:

1 $$\begin{aligned}&\dot{x}=-k_1x^2+k_4c-k_5ex+k_6d-k_8xy, \end{aligned}$$

2 $$\begin{aligned}&\dot{d}=k_5ex-(k_6+k_7)d,\end{aligned}$$

3 $$\begin{aligned}&\dot{e}=-k_5ex+(k_6+k_7)d,\end{aligned}$$

4 $$\begin{aligned}&\dot{c}=k_2fy-(k_3+k_4)c,\end{aligned}$$

5 $$\begin{aligned}&\dot{f}=-k_2fy+(k_3+k_4)c,\end{aligned}$$

6 $$\begin{aligned}&\dot{y}=k_1x^2-k_2fy+k_3c+k_7d+k_8xy, \end{aligned}$$

where the dot stands for the derivative with respect to *t* and the $k_i$ are positive rate constants. There are three conserved quantities defined by the total amounts of the substrate and the two enzymes *E* and *F*. These are $A=x+c+d+y$, $B=c+f$ and $C=d+e$. A situation where the amounts of both enzymes and the rates of both autophosphorylation reactions are small can be described using a Michaelis–Menten reduction. To do this, introduce new variables by means of the relations $k_1=\epsilon {\tilde{k}}_1$, $k_8=\epsilon {\tilde{k}}_8$, $c=\epsilon {\tilde{c}}$, $f=\epsilon {\tilde{f}}$, $d=\epsilon {\tilde{d}}$, $e=\epsilon {\tilde{e}}$ and $\tau =\epsilon t$. Substituting these relations in the above equations and dropping the tildes give

7 $$\begin{aligned}&x'=-k_1x^2+k_4c-k_5ex+k_6d-k_8xy,\end{aligned}$$

8 $$\begin{aligned}&y'=k_1x^2-k_2fy+k_3c+k_7d+k_8xy,\end{aligned}$$

9 $$\begin{aligned}&\epsilon d'=k_5ex-(k_6+k_7)d,\end{aligned}$$

10 $$\begin{aligned}&\epsilon e'=-k_5ex+(k_6+k_7)d,\end{aligned}$$

11 $$\begin{aligned}&\epsilon c'=k_2fy-(k_3+k_4)c,\end{aligned}$$

12 $$\begin{aligned}&\epsilon f'=-k_2fy+(k_3+k_4)c, \end{aligned}$$

where the prime stands for the derivative with respect to $\tau$. The motivations for this choice of rescaling are as follows. The first is that it can be used to find a smaller limiting system which is more accessible to mathematical analysis than the original one. The second is that the scaling of $k_1$ and $k_8$ ensures that competing effects are of a comparable order of magnitude in the limit and that this is conducive to the occurrence of interesting dynamical behaviour.

If we set $\epsilon =0$ in equations (7)–(12), the last four become algebraic. Combining these with the conservation laws and doing the usual algebra for Michaelis–Menten reduction leads to the relations $c=\frac{By}{K_{M1}+y}$ and $d=\frac{Cx}{K_{M2}+x}$ where $K_{M1}=\frac{k_3+k_4}{k_2}$ and $K_{M2}=\frac{k_6+k_7}{k_5}$. It follows that

13 $$\begin{aligned} x'=-k_1x^2+\frac{Bk_4y}{K_{M1}+y}-\frac{Ck_7x}{K_{M2}+x}-k_8{xy} \end{aligned}$$

while *y* satisfies an analogous equation. These two equations are equivalent because $x+y$ is a conserved quantity for $\epsilon =0$. Thus, the whole dynamics is contained in the single Eq. (13) in that case. When $C=0$ (no external kinase), the equation for *y* reduces (up to a difference of notation) to the equation (1) in Doherty et al. (2015). To make it clear that this is an equation for a single unknown, it is necessary to use the conserved quantity $A=x+y$. Thus, for $C=0$ the evolution equation for *y* is

14 $$\begin{aligned} y'=k_1(A-y)^2+k_8(A-y)y-\frac{Bk_4y}{K_{M1}+y}. \end{aligned}$$

## Analysis of the Model of Doherty et al.

In Doherty et al. (2015), the authors describe certain aspects of the dynamics of solutions of Eq. (14). Here we complement their analysis by giving rigorous proofs of some of these. Steady states of this equation are zeroes of the polynomial

15 $$\begin{aligned} p_3(y)&=[(k_1-k_8)y^2+(-2k_1A+k_8A)y+k_1A^2](K_{M1}+y)-Bk_4y\nonumber \\&= -(\alpha -1)y^3+[-K_{M1}(\alpha -1)+A(\alpha -2)]y^2\nonumber \\&\quad +[K_{M1}A(\alpha -2)+A^2-Bk_1^{-1}k_4]y+ K_{M1}A^2 \end{aligned}$$

where $\alpha =\frac{k_8}{k_1}$. Positive steady states of the evolution equations for *x* and *y* are in one-to-one correspondence with roots of this polynomial in the interval (0, *A*). Note that $p_3(0)>0$ and $p_3(A)<0$. If $k_1-k_8>0$, then $p_3$ must have one root with $x<0$ and one with $x>A$. Thus, it has exactly one root in the biologically relevant region. When $k_1-k_8<0$, there could be up to three roots in (0, *A*). Since no root can cross the endpoints of the interval, the number of roots counting multiplicity is odd for any values of the parameters. In biological terms, bistability is only possible when phosphorylation activates the enzyme. In the case of Lck, there are two phosphorylation sites of central importance for the regulation of the kinase activity, Y394 and Y505, whose phosphorylation is activatory and inhibitory, respectively. Thus, if we wanted to use this model to describe Lck with mutations targeting one of its phosphorylation sites, then to have a chance of bistability it is the inhibitory site Y505 which should be knocked out. This type of modification of Lck has been studied experimentally in Amrein and Sefton (1988). It was discovered that the mutated protein exhibits carcinogenic effects. This underlines the significance of the regulation of the activity of Lck through phosphorylation for the correct functioning of cells.

It will now be shown that there is a region in parameter space where three positive steady states exist.

### Theorem 1

If $\alpha >2$, $A^2k_1<Bk_4$, $k_8$ is sufficiently large and $K_{M1}$ is sufficiently small for fixed values of the other parameters, then Eq. (14) has three hyperbolic steady states, of which two are asymptotically stable and the other unstable.

### Proof

When three steady states exist, they must be simple zeros of $p_3$ and it follows that when ordered by the value of *y* the first and third steady states are stable, while the second is unstable. Each of these steady states is hyperbolic. Thus, to complete the proof of the theorem, it suffices to prove the existence of three steady states under the given assumptions. The condition for a steady state can be written in the form: $q_1(y)=q_2(y)$, where $q_1(y)=(A-y)[k_1A+(-k_1+k_8)y]$ and $q_2(y)=\frac{Bk_4y}{K_{M1}+y}$. Note that $q_2(y)<Bk_4$ for all $y\ge 0$. If $\alpha >1$, then $q_1$ has a local maximum when $y=y_1=\frac{(\alpha -2)A}{2(\alpha -1)}$. Assume that $\alpha >2$ so that $y_1>0$. Evaluating at the maximum gives $q_1(y_1)=\frac{k_8\alpha A^2}{4(\alpha -1)}$. By choosing $k_8$ large enough while keeping all other parameters fixed, we can ensure that this maximum is greater than $Bk_4$. It follows that $q_1(y_1)>q_2(y_1)$. Then, choosing $K_{M1}$ small enough while keeping all other parameters fixed and using the fact that $A^2k_1<Bk_4$ ensures that there is some $y_2<y_1$ with $q_1(y_2)<q_2(y_2)$. This implies that there are two roots of $p_3$ which are less than $y_1$ and these are simple. Under these conditions, $p_3$ has three positive roots in the interval (0, *A*) and so there exist three positive steady states. $\square$

**Fig. 1:**
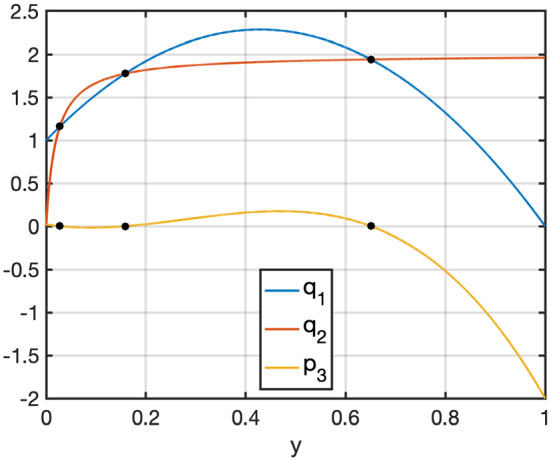
(Color figure online) Illustration of Theorem 1

This theorem and its proof are illustrated in Fig. 1 where we show $q_1$, $q_2$ and $p_3$ for parameters $A = 1$, $B = 2$, $k_1 = 1$, $k_4 = 1$, $k_8 = 8$, $\alpha = \frac{k_8}{k_1}$ and $K_{M1} = 0.02$ satisfying the assumptions in Theorem 1.

In fact, the three steady states arise in a single bifurcation. To prove this, we first need a result on cubic equations.

### Lemma 1

The polynomial $p(x)=ax^3+bx^2+cx+d$ has a triple root if and only if $b^3=27a^2d$ and $c^3=27ad^2$.

### Proof

If $x_*$ is a triple root, then $p(x_*)=p'(x_*)=p''(x_*)=0$. From the last of these equations, we can conclude that $x_*=-\frac{b}{3a}$. Substituting this in the other two equations gives $b^2=3ac$ and $b^3=\frac{9}{2}a(bc-3ad)$. Combining the last two equations gives $b^3=27a^2d$ and $c^3=27ad^2$. Suppose conversely that $b^3=27a^2d$ and $c^3=27ad^2$. Then, $bc=9ad$ and $abc=9a^2d$. Thus, $\frac{9}{2}(abc-3a^2d)=27a^2d$ and it follows that $b^3=\frac{9}{2}a(bc-3ad)$. Using $b^3=27a^2d$ then implies that $b^2=3ac$. With all this information, it can be checked directly that $x_*=-\frac{b}{3a}$ is a triple root of *p*. $\square$

### Theorem 2

The three steady states in Theorem 1 arise in a generic cusp bifurcation.

### Proof

To prove this, it will be shown that the parameters can be chosen so that the polynomial $p_3$ satisfies the conditions of Lemma 1. Assume that $K_{M1}<A$. Since $b^3=[-K_{M1}(\alpha -1)+A(\alpha -2)]^3$ we see that $b^3=(A-K_{M1})^3\alpha ^3+\cdots$ for $\alpha$ large and $b^3=(K_{M1}-2A)^3+\cdots$ for $\alpha \rightarrow 0$. Now $A-K_{M1}>0$ and $K_{M1}-2A<0$. Thus, if we consider $b^3$ as a function of $\alpha$ with the other parameters fixed it is an increasing function which takes on all values in the interval $[(K_{M1}-2A)^3,\infty )$. On the other hand, $a^2d=(\alpha -1)^2K_{M1}A^2$ and so $a^2d=K_{M1}A^2\alpha ^2+\cdots$ for $\alpha$ large and $a^2d=K_{M1}A^2+\cdots$ for $\alpha \rightarrow 0$. It follows that there exists an $\alpha _*$ for which $b^3=27a^2d$. In this way the first condition of Lemma 1 has been achieved. Since $a^2d$ is non-negative, there the same must be true of *b* and it follows that $\alpha _*>2$. Hence, $ad^2$ is negative and so in order to achieve the second condition of Lemma 1, it is enough to show that *c* can be given any prescribed negative value by choosing $k_4$ appropriately while fixing the other parameters. Note that *a*, *b* and *d* do not depend on $k_4$ so that the first condition remains satisfied. Since $\alpha _*>2$, the quantity *c* is positive for $\alpha =\alpha _*$ and $k_4$ sufficiently small. By increasing $k_4$, it can then be made to have any desired negative value. Thus, it can be ensured that the second condition is satisfied. Note that the point $x_*$ at which the bifurcation takes place does lie in the biologically relevant region (0, *A*) since there is one steady state in that region and $x_*$ is, neglecting multiplicity, the only one.

Next we note that the derivative of the mapping $(\alpha ,K_{M1},A,k_4)\mapsto (a,b,c,d)$ is always invertible for $\alpha >2$. Thus, by the inverse function theorem, we see that by varying the parameters arbitrarily we can vary the coefficients of the polynomial $p_3$ arbitrarily in a neighbourhood of the values for the triple root. Thus, we can choose two parameters so that the point with the triple root is embedded in a generic cusp bifurcation as defined in Kuznetsov (2010). More specifically, we can choose a mapping $(\beta _1,\beta _2)\mapsto (\alpha ,K_{M1},A,k_4)$ such that, after translating the coordinate *y* so that the bifurcation is at the origin, we have $(a,b,c,d)=(1,0,\beta _2,\beta _1)$. $\square$

Consider now the rescaled mass action system (7)–(12) in the case $C=0$. In this case, we can discard the equations for *d* and *e*. Moreover, we can use the conservation laws to discard the equations for *x* and *c* and replace these quantities in the right-hand sides of the equations for *y* and *f*. The result is

16 $$\begin{aligned}&y'=k_1(A-B-y+f)^2-k_2fy+k_3(B-f)+k_8xy,\end{aligned}$$

17 $$\begin{aligned}&\epsilon f'=-k_2fy+(k_3+k_4)(B-f). \end{aligned}$$

We now want to study the limit $\epsilon \rightarrow 0$ in these equations and show that solutions converge.

### Theorem 3

There is a choice of parameters such that the system (1)–(6) with $d=e=0$, $C=0$ and fixed values of *A* and *B* imposed has three steady states, of which two are asymptotically stable and the other a hyperbolic saddle. The three steady states arise in a generic cusp bifurcation. For arbitrary values of the parameters each solution converges to a steady state as $t\rightarrow \infty$. In particular, this system has no periodic solutions.

### Proof

It suffices to prove corresponding results for the system (16)–(17). The theorem can be proved using the results of Theorems 1 and 2 and geometric singular perturbation theory (GSPT) (Kuehn 2015). The important condition to be checked is that of normal hyperbolicity. It says that on the critical manifold, which is the zero set of the right-hand side in the equation for $f'$, the derivative of that right-hand side with respect to *f* should be nonzero. This is indeed the case since the derivative is $-k_2f-k_3-k_4<0$. It can be concluded that for each hyperbolic steady state of the Michaelis–Menten system, there is a nearby steady state of the mass action system which is hyperbolic within the invariant manifold of constant *A* and *B*. In addition, when the steady state of the Michaelis–Menten system is stable, the same is true of the corresponding steady state of the mass action system and when the steady state of the Michaelis–Menten system is unstable, the steady state of the mass action system is a saddle point whose stable manifold is one-dimensional. To obtain the statement about the convergence of general solutions to steady states, we compute the linearization of (16)–(17) which is

18 $$\begin{aligned} A=\left[ {\begin{array}{cc} 2k_1(f+y-A-B)-k_2f &{} 2k_1(f+y-A-B)-k_2y-k_3\\ -\epsilon ^{-1}k_2 f&{} -\epsilon ^{-1}[k_2y+(k_2+k_3)] \end{array}} \right] \end{aligned}$$

It is always the case that $A-y$ and $B-f$ are positive on the region of biological interest. Thus, the system is competitive. Every solution of a competitive two-dimensional system converges to a steady state (Smith 1995), and this completes the proof of the theorem. $\square$

To conclude this section, we consider the limiting case of the system (14) obtained by setting $k_1=0$. In this case only the phosphorylated form of the protein is catalytically active. Bistability for a system of this type was considered in Lisman (1985). If we continue to assume $C=0$, then $y=0$ is a steady state. Thus, in order to get bistability, we need to include that boundary steady state in the counting. With this understanding, we obtain an analogue of Theorem 1 for this case, where the condition on $\alpha$ is absent. The proof is strictly analogous to that of Theorem 1. To see what happens to Theorem 2 in this case, we need to replace $p_3$, which was got by division by $k_1$, by ${\tilde{p}}_3=y[k_8(-y+A)(y+K_{M1})-Bk_4]$. This polynomial has a triple root at the origin when $A=K_{M1}$ and $AK_{M1}=Bk_4$.

## Effect of an External Kinase

We next consider the case where the phosphorylated kinase is completely inactive, which can be modelled by setting $k_8=0$ in the model of the last section. This might be thought of as a model of the mutant of Lck where the activatory site Y394 is knocked out. It should, however, be noted that in reality the catalytic activity of this mutant, although much reduced, is not actually zero (Smith et al. 1993). In that case we have $k_1-k_8>0$ and, as mentioned above, there is only one positive steady state in the Michaelis–Menten system. Next, we will investigate the case where $k_1-k_8>0$ but an external kinase is present ($C>0$). It turns out that there is still only one steady state in the Michaelis–Menten system. For in any such steady state, we have

19 $$\begin{aligned} k_1x^2+k_8x(A-x)+\frac{Ck_7x}{K_{M2}+x}=\frac{Bk_4(A-x)}{K_{M1}+A-x}. \end{aligned}$$

Since the function on the left-hand side of this equation is monotone increasing on [0, *A*] and is zero for $x=0$, while the function on the right-hand side is monotone decreasing on [0, *A*] and is zero for $x=A$, these two functions are equal at a unique point $x\in (0,A)$. Thus, there cannot be more than one steady state in the Michaelis–Menten system with $k_8<k_1$. It turns out, however, that there can be more than one steady state in the corresponding mass action system, even in the case $k_8=0$.

Positive solutions of the mass action system with $k_8=0$ are in one-to-one correspondence with solutions of the following system obtained by using the conserved quantities to eliminate *d*, *c* and *y*.

20 $$\begin{aligned}&\dot{x}=-k_1x^2+k_4(B-f)-k_5ex+k_6(C-e),\end{aligned}$$

21 $$\begin{aligned}&\dot{e}=-k_5ex+(k_6+k_7)(C-e),\end{aligned}$$

22 $$\begin{aligned}&\dot{f}=-k_2f(A-B-C-x+e+f)+(k_3+k_4)(B-f). \end{aligned}$$

Define a polynomial by $p_6(x)=\sum _{i=0}^6a_ix^i$ with coefficients

23 $$\begin{aligned} a_6= & {} k_1^2k_2k_5^2, \end{aligned}$$

24 $$\begin{aligned} a_5= & {} 2k_1^2k_2k_5(k_6+k_7)+k_1k_2k_4k_5^2,\end{aligned}$$

25 $$\begin{aligned} a_4= & {} k_1k_2[-k_5^2((A+B)k_4-(k_4+2k_7)C-k_4(k_6+k_7))\nonumber \\&+\,k_1(k_6+k_7)^2+k_1k_5(k_6+k_7)]-k_1k_4(k_3+k_4)k_5^2,\end{aligned}$$

26 $$\begin{aligned} a_3= & {} k_1k_5(k_6+k_7)\{k_2[-2(A+B)k_4+(k_4+2k_7)C]\nonumber \\&-\,2(k_3+k_4)k_4\}+k_2k_4[k_5^2(Bk_4-k_7C)+k_1(k_6+k_7)^2],\end{aligned}$$

27 $$\begin{aligned} a_2= & {} k_2[-k_1k_4(A+B)(k_6+k_7)^2-k_4^2k_5(k_6+k_7)B\nonumber \\&+\,k_5^2(k_4A-(k_4+k_7)C-k_4(k_6+k_7))(Bk_4-k_7C)]\nonumber \\&-\,(k_3+k_4)k_4[k_1(k_6+k_7)^2+k_5^2k_7C],\end{aligned}$$

28 $$\begin{aligned} a_1= & {} k_2k_4k_5(k_6+k_7)[B(Ak_4-(k_4+k_7)C-k_4(k_6+k_7))\nonumber \\&+\,A(k_4B-k_7C)]-(k_3+k_4)k_4k_5(k_6+k_7)k_7C,\end{aligned}$$

29 $$\begin{aligned} a_0= & {} k_2k_4^2(k_6+k_7)^2AB. \end{aligned}$$

Define $x_{\mathrm{max}}$ to be the largest value of *x* satisfying the inequalities

30 $$\begin{aligned}&\frac{k_1}{k_4}x^2+\frac{k_5k_7Cx}{k_4(k_5x+k_6+k_7)}\le B,\end{aligned}$$

31 $$\begin{aligned}&x+\frac{k_1}{k_4}x^2+\left( 1+\frac{k_7}{k_4}\right) \frac{k_5Cx}{(k_5x+k_6+k_7)}\le A. \end{aligned}$$

Note that $x_{\mathrm{max}}$ depends continuously on the parameters.

### Lemma 2

For given positive values of *A*, *B* and *C*, positive steady state solutions of the system (1)–(6) with $k_8=0$ are in one-to-one correspondence with roots of the polynomial $p_6$ in the interval $(0,x_{\mathrm{max}})$.

### Proof

Note first that the equations for steady states of (1)–(6) are equivalent to the equations for steady states of (20)–(22) and that these in turn are equivalent to the equations

32 $$\begin{aligned}&k_5ex=(k_6+k_7)(C-e),\end{aligned}$$

33 $$\begin{aligned}&k_2f(A-B-C-x+e+f)=(k_3+k_4)(B-f),\end{aligned}$$

34 $$\begin{aligned}&k_1x^2=k_4(B-f)-k_7(C-e). \end{aligned}$$

Now suppose that (*x*, *d*, *e*, *c*, *f*, *y*) is a positive steady state. It follows from (32) that $e=\frac{(k_6+k_7)C}{k_5x+k_6+k_7}$ and combining this with (34) gives $f=B-(k_1/k_4)x^2-\frac{k_5k_7Cx}{k_4(k_5x+k_6+k_7)}$. Thus, we have solved for *e* and *f* in terms of *x*. Substituting this information in (33) and rearranging give the equation $p_6(x)=0$. Suppose conversely that *x* is a root of $p_6$ with $0<x<x_{\mathrm{max}}$. Define

35 $$\begin{aligned}&e=\frac{(k_6+k_7)C}{k_5x+k_6+k_7},\end{aligned}$$

36 $$\begin{aligned}&f=B-(k_1/k_4)x^2-(k_7/k_4)(C-e),\end{aligned}$$

37 $$\begin{aligned}&y=A-B-C-x+e+f. \end{aligned}$$

It follows from (30) that the quantity *f* defined by (36) is positive and from (31) that the quantity *y* defined by (37) is positive. It follows directly that (32) and (34) hold. The fact that *x* is a root of $p_6$ implies that (33) holds and hence that (*x*, *d*, *e*, *c*, *f*, *y*) is a positive solution of (1)–(6). $\square$

Consider now the real roots of $p_6$. They depend continuously on the parameters and their number is constant modulo two. Since $p_6(0)>0$ a root of $p_6$ cannot pass through zero. We claim that $x_{\mathrm{max}}$ can also never be a root of $p_6$. For if *x* were equal to $x_{\mathrm{max}}$ while satisfying the inequalities (31), then at least one of them would become an equality. In the first case *f* as defined by (36) would be equal to zero. This contradicts equation (33). In the second case *y* defined by equation (37) would be equal to zero. But then it follows from (4) that $c=0$ and from (6) that $x=0$, a contradiction. It can be concluded that the sign of $p_6(x_{\mathrm{max}})$ is independent of the parameters. To determine what the sign is it suffices to evaluate it for some particular values of the parameters. Choose $k_i=1$ for all *i*, $A=5$, $B=2$ and $C=3$. When $x=1$, we see that equality holds in (30), while the strict inequality holds in (31). Thus, in this case $x_{\mathrm{max}}=1$. Evaluating the coefficients in $p_6$ gives $a_6=1$, $a_5=5$, $a_4=8$, $a_3=-15$, $a_2=-43$, $a_1=-18$, $a_0=40$. Hence, $p_6(x_{\mathrm{max}})=-22<0$. It follows from the intermediate value theorem that $p_6$ has a least one root in each of the intervals $(0,x_{\mathrm{max}})$ and $(x_{\mathrm{max}},\infty )$. The number of sign changes of the coefficients in the polynomial is even and at most four. Thus, Descartes’ rule of signs implies that the number of positive roots is zero, two or four. The case with no positive roots has already been ruled out. Thus, there are two or four and at least one of them must be greater that $x_{\mathrm{max}}$. With the parameter values in the example, there are only two changes of sign, only two positive roots and we know that precisely one is less than $x_{\mathrm{max}}$. By continuity the number of roots in $(0,x_{\mathrm{max}})$ counting multiplicity is odd. It can only be one or three and we have already seen an example of parameters where it is one. In that case the system (1)–(6) admits precisely one positive steady state.

We will show that there also exist parameter values such that the system has three positive steady states. One approach would be to show that there are parameters for which there is a triple root in the desired interval and then perturb. In fact, we will show directly that there are parameters for which there are three roots in that interval, since that approach is simpler.

### Theorem 4

There is a choice of parameters for which the system (1)–(6) with $k_8=0$ has three positive steady states.

### Proof

Due to Lemma 2, it suffices to find parameter values for which the polynomial has three roots in the interval $(0,x_{\mathrm{max}})$. It follows from the preceding discussion that it is enough to show that the interval contains at least two roots. It turns out that it suffices to choose $A=6$, $B=20$, $C=2$, $k_i=1$ for all $i\ne 5$ and $k_5$ sufficiently large. With these choices, it follows that we get the following asymptotics for $k_5\rightarrow \infty$. $a_6=k_5^2+\cdots$, $a_5=k_5^2+\cdots$, $a_4=-20k_5^2+\cdots$ $a_3=18k_5^2+\cdots$, $a_2=-4k_5^2+\cdots$ where the terms not written explicitly are $o(k_5^2)$, as are the coefficients $a_1$ and $a_0$. It follows that $k_5^{-2}x^{-2}p(x)=q(x)+o(1)$, where $q(x)=x^4+x^3-20x^2+18x-4$. In the limit the inequalities defining the admissible interval become $x^2<18$ and $x+x^2<2$. By continuity it suffices to show that *q* has two roots in the interval (0, 1). This is true because $q(0)<0$, $q(1/2)>0$ and $q(1)<0$. $\square$

## Analysis of a Model for Wild-Type Lck

The results of the previous sections were related to situations in which one of the two key regulatory phosphorylation sites in a Src family kinase such as Lck is mutated. In the present section, we move to the case where both sites are present. The starting point for the discussion is the model introduced in Kaimachnikov and Kholodenko (2009). There four phosphorylation states of the kinase are included in the description. The first, denoted by $S_i$, is that where the inhibitory site is phosphorylated, while the activatory site is not. This form of the kinase shows no catalytic activity. *S*, $S_{a1}$ and $S_{a2}$ are the forms where neither site is phosphorylated, only the activatory site is phosphorylated and both sites are phosphorylated, respectively. All of these are catalytically active to some extent and can catalyse the transition $S\rightarrow S_{a1}$. The transitions $S\rightarrow S_i$ and $S_{a1}\rightarrow S_{a2}$ are catalysed by Csk. The transitions $S_i\rightarrow S$ and $S_{a2}\rightarrow S_{a1}$ are catalysed by one phosphatase, and the transitions $S_{a1}\rightarrow S$ and $S_{a2}\rightarrow S_i$ are catalysed by another phosphatase. Experimental results obtained in Hui and Vale (2014) indicate that some modifications of these assumptions may be needed to obtain a biologically correct model. In particular, it was found that Y505 in Lck undergoes autophosphorylation in trans, albeit with a much lower rate than Y394. The variants of the model of Kaimachnikov and Kholodenko (2009) which would be needed to take this into account will not be considered further in the present paper—the aim here is rather to see the variety of dynamical behaviour which this type of system can produce.

Let us introduce the following neutral notation for the quantities involved in the model, denoting the concentrations of *S*, $S_i$, $S_{a1}$ and $S_{a2}$ by $x_1$, $x_2$, $x_3$ and $x_4$, respectively. Then, $X=x_1+x_2+x_3+x_4$ is a conserved quantity. The reaction rate for the autophosphorylation is bilinear, the dephosphorylation of $S_{1a}$ is given by Michaelis–Menten kinetics, and the other reactions are assumed to be linear. Using the notations of Kaimachnikov and Kholodenko (2009) for the rate constants gives the system

38 $$\begin{aligned}&\dot{x}_1=-k_2x_1+k_1x_2+k_4\frac{x_3}{\beta +x_3}-k_3x_1(\delta x_1+x_3+x_4), \end{aligned}$$

39 $$\begin{aligned}&\dot{x}_2=k_2x_1-k_1x_2+k_7x_4,\end{aligned}$$

40 $$\begin{aligned}&\dot{x}_3=k_3x_1(\delta x_1+x_3+x_4)-k_4\frac{x_3}{\beta +x_3} +k_6x_4-k_5x_3,\end{aligned}$$

41 $$\begin{aligned}&\dot{x}_4=k_5x_3-(k_6+k_7)x_4. \end{aligned}$$

Before considering this system in the general case note that setting $x_2$, $x_4$, $k_2$ and $k_5$ to zero reduces this system to

42 $$\begin{aligned}&\dot{x}_1=k_4\frac{x_3}{\beta +x_3}-k_3x_1(\delta x_1+x_3),\end{aligned}$$

43 $$\begin{aligned}&\dot{x}_3=k_3x_1(\delta x_1+x_3)-k_4\frac{x_3}{\beta +x_3}. \end{aligned}$$

Either of the variables can be eliminated using the conserved quantity giving an equation which is, up to a difference in notation, exactly the equation of Doherty et al. discussed in previous sections. Only setting $k_2$ and $k_5$ to zero in (38)–(41) gives a partially decoupled system which is the product of the system of Doherty et al. (2015) with a hyperbolic saddle. It follows immediately from Theorem 1 that for suitable values of the parameters the system (38)–(41) admits at least three positive steady states, of which two are stable and hyperbolic and the third is a hyperbolic saddle.

We now return to the general system (38)–(41). In Kaimachnikov and Kholodenko (2009), the authors find a remarkable variety of dynamic behaviour in the system above which, after fixing a value of the conserved quantity, is of dimension three. They remark that there is a limiting case which gives rise to a system of dimension two which already exhibits a lot of this dynamics. To investigate this possibility, we define a new variable by $y=x_3+x_4$ and use it to replace $x_3$. In addition, we introduce rescaled parameters satisfying ${\tilde{k}}_5=\epsilon k_5$ and ${\tilde{k}}_6=\epsilon k_6$. Making these substitutions and discarding the tildes leads to the system

44 $$\begin{aligned} \dot{x}_1&=-k_2x_1+k_1x_2+k_4\frac{y-x_4}{\beta +y-x_4} -k_3x_1(\delta x_1+y), \end{aligned}$$

45 $$\begin{aligned} \dot{x}_2&=k_2x_1-k_1x_2+k_7x_4,\end{aligned}$$

46 $$\begin{aligned} \dot{y}&=k_3x_1(\delta x_1+y) -k_4\frac{y-x_4}{\beta +y-x_4}-k_7x_4, \end{aligned}$$

47 $$\begin{aligned} \epsilon \dot{x}_4&=k_5(y-x_4)-(k_6+\epsilon k_7)x_4. \end{aligned}$$

This is a fast-slow system with one fast and three slow variables. We have the conserved quantity $X=x_1+x_2+y$. In the limiting case $\epsilon =0$ Eq. (47) reduces to $y-x_4=\xi x_4$, where $\xi =\frac{k_6}{k_5}$. It follows that $x_4=\frac{1}{1+\xi }y$. Substituting this in (44) and (45) and using the conserved quantity give the following system of two equations:

48 $$\begin{aligned} \dot{x}_1&=-k_2x_1+k_1x_2+k_4\frac{\xi (X-x_1-x_2)}{\beta (\xi +1)+\xi (X-x_1-x_2)} \nonumber \\&\qquad -k_3x_1[(X-x_1-x_2)+\delta x_1], \end{aligned}$$

49 $$\begin{aligned} \dot{x}_2&=k_2x_1-k_1x_2+k_7\frac{1}{\xi +1}(X-x_1-x_2). \end{aligned}$$

In the terminology of GSPT, this is the restriction of the system to the critical manifold. This critical manifold is normally hyperbolic and stable since the partial derivative of the right-hand side of (47) with respect to $x_4$ evaluated at $\epsilon =0$ is negative. This allows us to transport information about stability and bifurcations from steady states of the two-dimensional system to steady states of the full system. Positive steady states of the full system with a given value of *X* are in one-to-one correspondence with positive steady states of (48)–(49) with $x_1+x_2<X$. At steady state equation (49) can be used to express $x_2$ in terms of $x_1$ and substituting this in (48) shows that $x_1$ is a root of a cubic polynomial which is not identically zero. Thus, the system (48)–(49) has at most three steady states.

It will be shown that the system (48)–(49) admits periodic solutions which arise in a Hopf bifurcation and homoclinic orbits. In order to do this it suffices to show that this system admits a generic Bogdanov–Takens bifurcation (Kuznetsov 2010). By saying that the bifurcation is generic, we mean that it satisfies the conditions BT.0, BT.1, BT.2 and BT.3 of Kuznetsov (2010). Then, the desired results follow from Theorem 8.5 of Kuznetsov (2010) and the analysis of the normal form of the bifurcation preceding that theorem. Let $J(x_1,x_2)$ be the linearization of the system (48)–(49) about the point $(x_1,x_2)$. Finding a bifurcation point where the condition BT.0 is satisfied means finding a point $(x_1,x_2)$ and a choice of the parameters of the system so that $J(x_1,x_2)$ has a double zero eigenvalue but is not itself zero. If the right-hand sides of equations (48) and (49) are denoted by $f_1$ and $f_2$, this means solving the system of four equations given by the vanishing of $f_1$, $f_2$, $\mathrm{tr} J$ and $\det J$. The general strategy is to choose all but four of the parameters and use the four equations to solve for the rest. An obstacle to this is that the quantities resulting from this process might fail to be positive. This obstacle was overcome by trial and error.

The equations for steady states can be written in the following form:

50 $$\begin{aligned} k_4&=\frac{[k_2x_1-k_1x_2+k_3x_1(X-x_1-x_2+\delta x_1)] [\beta (\xi +1)+\xi (X-x_1-x_2)]}{\xi (X-x_1-x_2)},\end{aligned}$$

51 $$\begin{aligned} k_7&=\frac{(\xi +1)(-k_2x_1+k_1x_2)}{X-x_1-x_2}. \end{aligned}$$

The linearization is

52 $$\begin{aligned} J=\left[ {\begin{array}{cc} -k_2-\phi (\beta )-k_3(X-2x_1-x_2+2\delta x_1) &{} k_1-\phi (\beta )+k_3x_1\\ \eta &{} -\omega \end{array}} \right] \end{aligned}$$

Here, we have introduced the auxiliary quantities $\eta =k_2-\frac{k_7}{\xi +1}$, $\omega =k_1+\frac{k_7}{\xi +1}$ and $\phi (\beta )=\frac{k_4\xi (\xi +1)\beta }{[\beta (\xi +1)+\xi (X-x_1-x_2)]^2}$. Suppose that we have a Bogdanov–Takens bifurcation. Since the trace is zero, we have that the first element in the first row of the Jacobian must be equal to $\omega$. Hence,

53 $$\begin{aligned} \phi (\beta )+\omega +k_2=-k_3[X-2(1-\delta )x_1-x_2]. \end{aligned}$$

It follows that

54 $$\begin{aligned} k_1-\phi (\beta )+k_3x_1 =\omega +k_1+k_2+k_3[X-(1-2\delta )x_1-x_2]. \end{aligned}$$

Since the determinant is zero, we have

55 $$\begin{aligned} \omega ^2+\eta [\omega +k_1+k_2+k_3(X-x_1-x_2+2\delta x_1)]=0. \end{aligned}$$

Choose $X=\frac{3}{2}$, $x_1=1$, $x_2=\frac{1}{4}$, $k_1=8$, $k_2=1$, $\delta =\frac{1}{6}$, $\xi =1$. It follows from (51) that $k_7=8$. Putting this into (55) gives $k_3=\frac{324}{7}$. It then follows from (53) that $\phi (\beta )=\frac{44}{7}$. On the other hand, (50) implies that $k_4=\frac{128}{7}(8\beta +1)$. Combining this with the definition of $\phi$ shows that $\beta =\frac{11}{936}$. Finally, we compute $k_4=\frac{16384}{819}$. The conclusion is that with the given choices, there is exactly one solution for the remaining parameters $(k_7,k_3,\beta ,k_4)$ such that the system satisfies the condition BT.0 for a Bogdanov–Takens bifurcation at the chosen point with coordinates $(x_1,x_2)=\left( 1,\frac{1}{4}\right)$. At this point the linearization is of the form:

56 $$\begin{aligned} J=\left[ {\begin{array}{cc} 12&{} 48\\ -3&{} -12 \end{array}} \right] . \end{aligned}$$

When talking about a Bogdanov–Takens bifurcation, we need a system depending on two parameters. In our example, we choose these to be $\delta$ and $k_3$ and consider all other parameters in the system as fixed

As will now be explained, a calculation shows that conditions BT.1, BT.2 and BT.3 are also satisfied so that this is a generic Bogdanov–Takens bifurcation. For this purpose, it is convenient to transform to coordinates $y_1=-\frac{1}{3} x_2+\frac{1}{12}$ and $y_2=x_1+4x_2-2$ adapted to the eigenvectors of *J*. Then, $\dot{y}_i=(J_0y)_i+Q_i(y)+O(|y|^3)$, where $J_0$ is in Jordan form and the $Q_i$ are quadratic. In the notation of Kuznetsov (2010), the elements of $Q_1$ and $Q_2$ are denoted by $a_{ij}$ and $b_{ij}$, respectively. In the present example, it turns out that $a_{20}=0$ and in that case BT.1 and BT.2 are the conditions that $b_{11}\ne 0$ and $b_{20}\ne 0$. A lengthy calculation shows that $b_{20}=-81W+168k_3>0$ and $b_{11}=-9W+7k_3>0$, where $W=\frac{256\beta k_4}{(8\beta +1)^3}$. Here, we use the values of the parameters at the bifurcation point. The condition BT.3 is that the linearization $J_T$ of the mapping $(x_1,x_2,\delta ,k_3)\mapsto (f_1,f_2,\mathrm{tr}J,\det J)$ at the bifurcation point is non-singular. This matrix is

57 $$\begin{aligned} J_T=\left[ {\begin{array}{cccc} 12&{}\quad 48&{}\quad -k_3 &{}\quad -\frac{5}{12}\\ -3&{}\quad -12&{}\quad 0 &{}\quad 0\\ -W+\frac{5}{3} k_3&{}\quad -W+k_3&{}\quad -2k_3&{}\quad \frac{5}{12}\\ 9W-17k_3&{}\quad 9W-12k_3&{}\quad 24 k_3&{}\quad -2 \end{array}} \right] \end{aligned}$$

and $$\det J_T=-6k_3^2-\frac{27Wk_3}{4}\ne 0$$.

When a generic Bogdanov–Takens bifurcation is present in a dynamical system, then there are always generic Hopf bifurcations nearby. The periodic solutions which arise in these Hopf bifurcations are hyperbolic and may be stable (supercritical case) or unstable (subcritical case). We may correspondingly call the Bogdanov–Takens bifurcation super- or subcritical, and it turns out that these two cases are distinguished by the relative sign of $b_{20}$ and $b_{11}$. In the present case, the signs of these two coefficients are equal and the bifurcation is subcritical. Hence, the periodic solutions are unstable. In comparison with the phase portrait given in Kuznetsov (2010), which corresponds to the supercritical case, the direction of the flow is reversed. For the parameter values for which the bifurcation takes place, the cubic polynomial for $x_1$ has a double root at $x_1=1$ and must therefore have a factor $(x_1-1)^2$. Carrying out this factorization allows a third root to be calculated explicitly. The result is

58 $$\begin{aligned} p(x_1)=\frac{3}{728}(2457x_1-2924)(x_1-1)^2. \end{aligned}$$

The additional root is $x_1=\frac{2924}{2457}$ and at the corresponding steady state $x_2=\frac{995}{4914}$. At that point the trace of the linearization is negative and the determinant positive. Hence, this steady state is stable.

Some of these results will now be collected in a theorem.

### Theorem 5

There are parameter values for which the system (48)–(49) has a generic Bogdanov–Takens bifurcation. In particular, there are nearby parameter values for which it has an unstable periodic solution and ones for which it has a homoclinic orbit. In the case where there is an unstable periodic solution with parameter values sufficiently close to those at the bifurcation point, there are also two stable steady states and one saddle point.

The structural stability of the bifurcation and the fact that the limit used to obtain this system is normally hyperbolic imply that these features can be lifted to the system (38)–(41). In more detail, note first that this system is equivalent by rescaling to the system (44)–(47). Moreover, we can concentrate on a fixed value of the conserved quantity *X*. Thus, it remains to consider a limit from a three-dimensional system to a two-dimensional one. Restricting to the slow manifold we get a regular limit of two-dimensional systems. For $\epsilon =0$ the mapping $(x_1,x_2,\delta ,k_3)\mapsto (f_1,f_2,\mathrm{tr}J,\det J)$ has full rank and a zero at the bifurcation point. It follows from the implicit function theorem that it has a unique zero near the bifurcation point for $\epsilon$ small. This is a point where BT.0 is satisfied. By continuity BT.1, BT.2 and BT.3 remain satisfied for $\epsilon$ sufficiently small and the bifurcation remains subcritical. Thus, the features listed in Theorem 5 are also seen in the system on the slow manifold. This implies immediately that there is a homoclinic orbit in the full system. The hyperbolic periodic solution in the slow manifold is also hyperbolic as a solution of the full system. It is of saddle type with there being both solutions which converge to it for $t\rightarrow +\infty$ and solutions which converge to it for $t\rightarrow -\infty$. If it could be shown that the limiting system admits a stable periodic solution for some values of the parameters, it could be concluded that the full system does so too. We have not been able to prove the existence of stable periodic solutions of the limiting system. To see how such a stable solution might occur, consider the predator–prey model of Bazykin discussed in Kuznetsov (2010). It has two subcritical Bogdanov–Takens bifurcations and a stable periodic solution in a distant part of the phase space.

It turns out that it is possible to find an extension of the explicit Bogdanov–Takens point to an explicit two-parameter family of steady states, including a one-parameter family of points where the eigenvalue condition for a Hopf bifurcation is satisfied. The parameters are the determinant $\sigma$ and the trace $\tau$ of *J* at the steady state. This family is obtained by fixing the same quantities as in the original case, including the coordinates $(x_1,x_2)$ of the steady state and computing the parameters

59 $$\begin{aligned} k_3=\frac{324+4\sigma +36\tau }{7},\ \ \beta =\frac{132+5\sigma +24\tau }{11232+120\sigma +1248\tau } \end{aligned}$$

and

60 $$\begin{aligned} k_4=\frac{(384+5\sigma +45\tau )(1536+20\sigma +180\tau )}{21(1404+15\sigma +156\tau )}. \end{aligned}$$

**Fig. 2:**
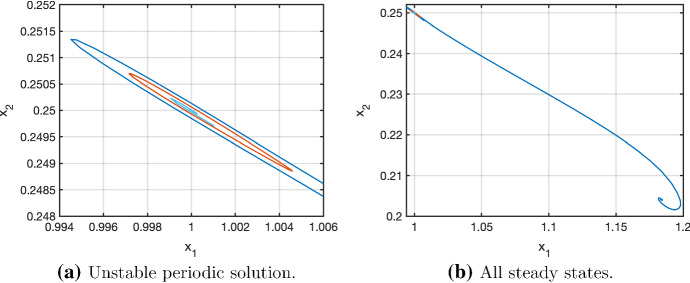
Phase diagram for $\sigma =1$, $\tau =-0.02$ (Color figure online)

The Hopf points are those with $\sigma >0$ and $\tau =0$. These formulae are helpful in finding parameter values for which the system admits an unstable periodic solution. A solution of this type is illustrated in Fig. 2 in red for the case $\sigma =1$, $\tau =-0.02$. In Fig. 2a, we show neighbouring solutions of the unstable periodic solution which move away from it (inward and outward spirals). A larger part of the phase space is shown in Fig. 2b where the Bogdanov–Takens point and the distant stable steady state are shown.

## Comparison with Some More Elaborate Models

In this section, the results of this paper will be put into a wider context by comparing the models analysed here with some more complicated models in the literature which arise when studying specific biological phenomena. One of the most exciting recent developments in medicine is immune checkpoint therapies for cancer (Robert 2020). Immune checkpoint molecules such as CTLA4 and PD-1 can result in the deactivation of T cells under certain circumstances, and this is exploited by cancer cells to evade attacks by the immune system. Antibodies to the immune checkpoint molecules can prevent this and thus be used in cancer therapies. This type of therapy has had remarkable success in curing some cancers. On the other hand, although these therapies could in principle work for all types of cancer, in practice they only work for some cancers, notably metastatic melanoma, and even in the most favourable cases for only a certain percentage of patients. It is important to obtain a better understanding of the molecular mechanisms of these therapies, so as to explain in which cases they are effective and, hopefully, to improve them so as to increase the range of their efficacy.

Up to now the most effective types of immune checkpoint monotherapy are those involving PD-1. In this context, it is important to understand how the activation of PD-1 leads to suppression of T cell activity. This has been studied experimentally in Hui et al. (2017). The main conclusion of that work is that the inhibition of T cell activity caused by PD-1 is due less to a decrease in signalling via the T cell receptor than to a decrease in the second signal coming from CD28. This leads to a certain mechanistic model of how the influence of PD-1 is exerted. In an effort to obtain a better understanding of the mechanisms involved, a mathematical model was introduced in Arulraj and Barik (2018). Simulations of that model gave results agreeing well with the results of Hui et al. (2017) and at the same time suggesting an additional path by which PD-1 can influence T cell signalling. In the path highlighted in Hui et al. (2017), activation of Lck plays an important role. The suggestion in Arulraj and Barik (2018) is that this change in the activation state of Lck could have an indirect influence via phosphorylation of molecules downstream of the T cell receptor and CD28. The model of Arulraj and Barik (2018) consists of several modules. One of these describes the activation of Lck and plays a central role.

In the context of their model of Lck, regulation Arulraj and Barik (2018) cite a model given in Rohrs et al. (2016). The latter includes complexes which are intermediates in the autophosphorylation reactions. This would correspond in the case with one phosphorylation site to replacing the reaction 2X$\rightarrow$X+Y by the reactions 2X$\rightarrow$X${}_2$ $\rightarrow$X+Y, where X${}_2$ is the complex formed when two molecules of X bind to each other. It also includes certain complexes of Lck with Csk which are analogous to XF in the basic model introduced in Sect. 2. According to Rohrs et al. (2016), the inclusion of these complexes was necessary to obtain a good agreement between the results of simulations and the experimental data of Hui and Vale (2014). The model of Rohrs et al. (2016) includes no phosphatases and so it is clear that in that case the evolution must converge to the state where only the unique maximally phosphorylated state is present. The non-trivial characteristics of the evolution have to do with the way in which the solution approaches that state.

One difference of the model of Arulraj and Barik (2018) compared to that of Kaimachnikov and Kholodenko (2009) is that it includes five forms of Lck rather than four. The model contains two different forms of doubly phosphorylated Lck which are supposed to differ by the order in which the two sites were phosphorylated. The issue of the order of phosphorylation is mentioned in Hui and Vale (2014), but we are not aware of any justification for including the fifth form in the model. It is stated in Arulraj and Barik (2018) that the model includes autophosphorylation of Lck, but in the equations the dependence on the concentrations of the different forms of Lck is everywhere linear and this does not seem to be consistent.

In Schulze (2014), the author discusses the model of Kaimachnikov and Kholodenko (2009) and the alternative where the Michaelis–Menten term in that model is replaced by a linear one. When that simplification is made, bistability is eliminated. The author presents unpublished data of Acuto and Nika which addresses the issue of bistability in Lck experimentally. The idea is that if bistability was present, the distribution of the measurements of certain quantities in a population should be bimodal, i.e. the graph should exhibit two maxima. In these data, most (but not all) of the graphs have a unique maximum and this is taken as evidence that there is no bistability in the system. However, no detailed justification for this conclusion is given. The significance of this conclusion is that if bistability were present in the biological system, this would mean that the simplified model would not be sufficient. In Schulze (2014), the simplified model is used. The advantage is that there are fewer parameters in the simplified model and that their values can be more strongly constrained by experimental data.

Another biological phenomenon where Lck plays a central role is that of T cell activation. It will now be discussed how Lck has been modelled in the literature on that subject. One of the first and most important steps in T cell activation is the phosphorylation of the ITAMs (immunoreceptor tyrosine-based activation motifs) of the T cell receptor complex. The most important kinase carrying out this process is Lck. In one successful model of early T cell activation (François et al. 2013), Lck is not one of the chemical species included in the model. In the process of ITAM phosphorylation, Lck is treated as an external kinase whose activity is represented by a rate constant. It was proved in Rendall and Sontag (2017) that this model can exhibit more than one steady state. The model of (François et al. 2013) is a radical simplification of a more extensive one introduced in Altan-Bonnet and Germain (2005). In the latter model, activated Lck is one of the chemical species included. It takes part in many reactions where it binds to a complex X containing the T cell receptor and some other molecules and then phosphorylates some element of the complex. The kinetics of these reactions is extended Michaelis–Menten. Other forms of Lck play a role in mechanisms represented in this model, but they do not appear explicitly. Another model implementing some of the same mechanisms was presented in Lipniacki et al. (2008). It includes four forms of Lck arising from phosphorylation at Y394 and the serine S59. The serine phosphorylation may have an important role to play in T cell activation but will not be considered further here. The tyrosine phosphorylation is supposed to occur by autophosphorylation in trans, but the Lck molecules responsible for the catalysis are supposed to belong to a different population to those being phosphorylated. The former population is treated as external and so no nonlinearity arises from this process. The model of Lipniacki et al. (2008) exhibits bistability.

## Conclusions and Outlook

In this paper, we proved that the model of Doherty et al. (2015) of an enzyme with a single site subject to autophosphorylation in trans can exhibit bistability. This improves on the simulations in Doherty et al. (2015) showing this type of behaviour for specific parameter values by identifying a large part of parameter space where it occurs. We also show that in the context of this model multiple steady states can only occur when the phosphorylation increases the activity of the enzyme. It is shown that in a case where phosphorylation decreases the activity of the enzyme multiple steady states can also occur but this requires a more complicated model with an external kinase which is operating well away from the Michaelis–Menten limit.

We related the models studied in this paper to other models involving Lck which have been applied in the literature to describe particular biological phenomena. It is of interest to consider the possible biological meaning of the results of this paper. Switches arising through bistability are a well-known phenomenon in biology and the bistability found in the regulation of Lck might be of importance for immunology as a mechanism by which the activity of immune cells is switched off or on in certain circumstances. As discussed in the last section, it seems unclear on the basis of experimental evidence whether bistability due to the properties of Lck occurs in biologically interesting circumstances. We are not aware that oscillations in the activation of Lck have been observed experimentally. The biological significance of those periodic solutions whose existence we proved is limited by their instability.

This paper is a preliminary exploration of dynamical features of models for Lck involving autophosphorylation. At this point, it is appropriate to think about what biological issues could be illuminated by continuing these investigations. A question of great biological and medical interest, already mentioned in the last section, is that of the mechanism by which ligation of the receptor PD-1 leads to the suppression of the activity of T cells. (For a recent review of this topic, see Patsoukis et al. 2020.) Normally the activation of a T cell requires both a signal from the T cell receptor and a second signal from CD28. Both of these receptors get phosphorylated. (In the case of the T cell receptor, it is rather the associated proteins CD3 and the $\zeta$-chain which are phosphorylated.) A question which is apparently still controversial is whether the main effect of PD-1 activation is dephosphorylation of the T cell receptor or that of CD28. The conclusion of Hui et al. (2017) is that it is CD28, but this has been disputed in Mizuno et al. (2019), where it has been suggested that this finding of Hui et al. (2017) was an artefact of using a cell-free system and that in reality it is dephosphorylation of the T cell receptor which is the most important consequence of the activation of PD-1. This indicates that better understanding of these phenomena is necessary. Arulraj and Barik (2018) claim that their model can reproduce the results of Hui et al. (2017). Could that model, or a related one, reproduce the results of Mizuno et al. (2019)?

There is a wide consensus that, whatever the targets of dephosphorylation resulting from the activation of PD-1, the phosphatase which carries it out is SHP-2. There is one caveat here since it was observed in Rota et al. (2018) that PD-1 can have an inhibitory effect on T cells in the absence of SHP-2. This issue deserves further investigation. Another interesting question is that of the way in which Lck interacts with SHP-2 and PD-1. When PD-1 is fully activated, it is phosphorylated at two sites. These provide binding sites for SHP-2. The phosphorylation of PD-1 is catalysed primarily by Lck (Hui et al. 2017). SHP-2 can dephosphorylate PD-1 and thus promote its own unbinding. This effect is opposed by Lck. Here, there is an incoherent feed-forward loop (Alon 2006). On the one hand, Lck causes phosphorylation of PD-1 by a direct route and on the other hand, it causes its dephosphorylation by an indirect route. These interactions are described by one of the modules in the model of Arulraj and Barik (2018). They are sufficiently complex that it would be desirable to carry out a deeper mathematical analysis of their dynamics.
